# Report of the Prime Minister's Committee

**Published:** 1918-05-11

**Authors:** 


### THE POSITION OF SCIENCE IN EDUCATION.

### Report of the Prime Minister's Committee.

### I. SUBJECTS TO BE TAUGHT.

Committees on Education are just now as plenty as blackberries. The Report of the Committee presided over by Sir J. J. Thomson, O.M., P.R.S., is certainly the best that has appeared thus far. It is of very wide scope, and reviews our whole system of education, from the elementary school and the preparatory school to the highest degrees of the universities. The amount of evidence taken and of material taken into consideration is enormous, and the members of the Committee must have worked very hard to produce in no more than twenty months a Report so careful, so well-digested, so comprehensive, so complete, and finally, so little open to criticism that even *The Times*, committed as it is up to the chin to the defence of the existing scheme of classical education in our public schools, is compelled to greet the Report with a grudging approval. Apart from its objection that the majority of the Committee are men of science, and the sneer that there is nothing like leather, and apart from the ludicrous comparison of compulsory science to compulsory Greek, *The Times*, which evidently tries to ban the Report, is constrained to accord to it a modified and grudging blessing. If the Report now issued be read, says *The Times*, and its recommendations carried out . . . it should prove a useful milestone on the road to reform. We will venture a little further, and declare that if the recommendations of the Report are carried out, they will obviate the need of further reform for many a long year to come. Improvement in details will no doubt continue to be required; but as far as intellectual education is concerned, as far as training of the intellect alone is in question, apart from the moral aspect of education, which was outside the terms of reference of the Committee, the Report lays down the main lines of education with something approaching to finality. Nothing in this world is made to last for ever; but if the recommendations of the Committee were carried out, there would be an end for generations to come to the perpetual tinkering with the groundwork of education, and schools, universities, and the Education Department would know what they have to do, and would be able to get on with their proper work of education without being compelled to break off at frequent intervals and dislocate their work for the introduction of some new principle, some new method, or some new class of subjects.

The part of the Report that interests us specially is, of course, that devoted to medical education; but so thoroughly systematised is the Report, and so coherently do all the recommendations hang together, that it is impossible to consider the part that discusses medical education without considering also that which concerns the general education that should precede entrance upon medical studies.

The fundamental proposal of the Report is that instruction in the facts, and especially in the methods, of science, by which is meant the whole group of natural sciences, as distinguished from mathematics, etc., is necessary to a liberal education; and that while a knowledge of Greek is not necessarily a part of a liberal education, a knowledge of natural science is. A knowledge of natural science is of course useful, but the Committee very rightly does not base its advocacy of science upon the score of its utility. Science has, as the Committee points out, several kinds of educational value. "It can arouse and satisfy the element of wonder in our natures. As an intellectual exercise it disciplines our powers of mind. Its utility and applicability are obvious. It quickens and cultivates directly the powers of observation. It teaches the learner to reason from facts that come under his own notice. By it, the power of rapid and accurate generalisation is strengthened. Without it, there is a real danger of the mental habit of method and arrangement never being acquired . . . It has often been noticed that boys when they begin to learn science receive an intellectual refreshment which makes a difference even to their literary work . . . A nation thoroughly trained in scientific method and stirred with an enthusiasm for penetrating and understanding the secrets of nature, would no doubt reap a rich material harvest of comfort and prosperity; but its truest reward would be that it would be fitted by 'an ample and generous education to perform justly, skilfully, and magnanimously the offices both private and public of peace and war.'"

The teaching of science should begin at the beginning of education, in the elementary and preparatory schools. Of course, at this stage, the teaching of science should be elementary and preparatory. It should consist of nature study of the most practical character, full use being made of the school garden, and should be accompanied by manual training, both for the sake of the discipline it supplies for hand and eye, and as an introduction to laboratory work. Properly conducted, the instruction should familiarise the pupil with the notion of measurement, and train him in habits of accuracy. Methods of measurement may be applied to, and elementary scientific conceptions derived from, exercises in wood-work, simple metal work, gardening, and cookery; thus the science teaching, gardening, drawing, and other branches of hand work will help each other.

When the secondary school is reached, the teaching of science should be developed and elaborated on the groundwork already established in the elementary or preparatory school. Specialisation is not to begin before the age of sixteen, and up to that age the education should be in English, including history and geography, in languages other than English, and in mathematics and natural science. We think the inclusion of history and

**THE POSITION OF SCIENCE IN EDUCATION** (*cont.*)

geography in English is illogical, and that the separation of geography from physiography, and the exclusion of the latter can scarcely be defended; moreover, we would go further than the Committee in deprecating the necessary inclusion of languages other than English. There are boys, and girls also, who cannot learn a second language, and this inability is especially apt to occur in those who have a bent for science. At this stage options should begin, and while those who have linguistic ability should be encouraged to cultivate it, those who have none should have the opportunity of spending their time profitably instead of wasting it upon a study in which they will never be successful.

A practical and beneficial consequence of the establishment of a general course for all boys up to the age of about sixteen would be the abolition of the division into " sides " now so usual in the larger schools. The last thing the Committee would suggest is that there should be for all boys, even in the same school, and during the stage ending at sixteen, one cast-iron curriculum; but as long as the division into " sides " persists, not only is a bias inevitably given to a boy's education, involving sometimes the sacrifice of his abilities and tastes, but it is very difficult to prevent the intellectual strength of the school being concentrated on one side. This is notoriously what happens at present, the side on which the ability is concentrated being, of course, the classical side; and then it is contended that as the classical side turns out the cleverest boys it must provide the best training, the fact being, of course, that it turns out the cleverest boys because it takes care to take in the cleverest boys.

When the age of sixteen is reached, boys should present themselves for the First School Examination, for which some better title could surely be found, for the boys must already have sat for many school examinations. This First School Examination, whatever its title, should form a portal of entrance to every university and every profession. The attainment of a sufficient standard in this examination should stand in lieu of Responsions at Oxford, of the Previous Examination at Cambridge, of matriculation at the London and other universities, and *inter alia* of the many examinations accepted by the General Medical Council for entrance into the medical profession.

The advantage of such a system to students entering the medical profession, and to the profession itself, would be almost incalculably great. At the present time the examinations accepted by the General Medical Council, as furnishing sufficient evidence of a general liberal education to justify admission into the medical profession, are not only very numerous, but also very widely different in value, and very many of them may be passed with distinction by boys who have never studied any subject of natural science for an hour. The first professional examination to be passed by the medical student after entering the university or medical school is an examination in natural science, that is, in physics, chemistry, and biology. The medical curriculum extends over a minimum of five years, and students who enter on their medical studies utterly ignorant of natural science must cram into those five years sufficient study of natural science to enable them to pass the first professional examination. In this they not only stand at a great disadvantage in comparison with those who have been thoroughly grounded in natural science from their earliest school years, but also they cannot possibly reap from the years of their studentship as much knowledge of the medical sciences proper as they ought to do, and as they would do if they entered the profession after a proper preparation for it. Those who cram up in one year sufficient verbal knowledge of the preliminary medical sciences cannot possibly possess any real knowledge of these sciences, and are fettered and hampered throughout the whole of their after-life by the imperfection of their knowledge of these subjects. By the system proposed in the Report we are considering, the embryo medical student will have been familiarised with natural science and its methods from his earliest entrance into an elementary or preparatory school. At his secondary school he will have continued this familiarity, and will have broadened and deepened his knowledge of natural science and its methods until the age of sixteen. He will then have a good general foundation of knowledge of physics, chemistry, and biology, but his knowledge will be of a general character, as part of a liberal education, and will have no special application to medicine until the age of sixteen and the First School Examination are left behind.

(*To be continued.*)

